# The functional false discovery rate with applications to genomics

**DOI:** 10.1093/biostatistics/kxz010

**Published:** 2019-05-28

**Authors:** Xiongzhi Chen, David G Robinson, John D Storey

**Affiliations:** Lewis-Sigler Institute for Integrative Genomics, Princeton University, Princeton, NJ 08544, USA

**Keywords:** eQTL, FDR, Functional data analysis, Genetics of gene expression, Kernel density estimation, Local false discovery rate, Multiple hypothesis testing, *q*-value, RNA-seq, Sequencing depth

## Abstract

The false discovery rate (FDR) measures the proportion of false discoveries among a set of hypothesis tests called significant. This quantity is typically estimated based on *p*-values or test statistics. In some scenarios, there is additional information available that may be used to more accurately estimate the FDR. We develop a new framework for formulating and estimating FDRs and *q*-values when an additional piece of information, which we call an “informative variable”, is available. For a given test, the informative variable provides information about the prior probability a null hypothesis is true or the power of that particular test. The FDR is then treated as a function of this informative variable. We consider two applications in genomics. Our first application is a genetics of gene expression (eQTL) experiment in yeast where every genetic marker and gene expression trait pair are tested for associations. The informative variable in this case is the distance between each genetic marker and gene. Our second application is to detect differentially expressed genes in an RNA-seq study carried out in mice. The informative variable in this study is the per-gene read depth. The framework we develop is quite general, and it should be useful in a broad range of scientific applications.

## 1. Introduction

Multiple testing is now routinely conducted in many scientific areas. For example, in genomics, RNA-seq technology is often utilized to test thousands of genes for differential expression among two or more biological conditions. In expression quantitative trait loci (eQTL) studies, all pairs of genetic markers and gene expression traits can be tested for associations, which often involves millions or more hypothesis tests. The false discovery rate (FDR, Benjamini and Hochberg, 1995) and the *q*-value (Storey, 2002, 2003) are often employed to determine significance thresholds and quantify the overall error rate when testing a large number of hypotheses simultaneously. Therefore, improving the accuracy in estimating FDRs and *q*-values remains an important problem.

In many emerging applications, additional information on the status of a null hypothesis or the power of a test may be available to help better estimate the FDR and *q*-value. For example, in eQTL studies, gene–single nucleotide polymorphism (SNP) basepair distance informs the prior probability of association between a gene–SNP pair, with local associations generally more likely than distal associations (Brem *and others*, 2002; Doss *and others*, 2005; Ronald *and others*, 2005). A second example comes from RNA-seq studies, for which per-gene read depth informs the statistical power to detect differential gene expression (Tarazona *and others*, 2011; Cai *and others*, 2012) or the prior probability of differential gene expression (Robinson *and others*, 2015). Genes with more sequencing reads mapped to them (i.e., higher per-gene read depth) have greater ability to detect differential expression or may be more likely to be differentially expressed than do low depth genes.

**Fig. 1.**
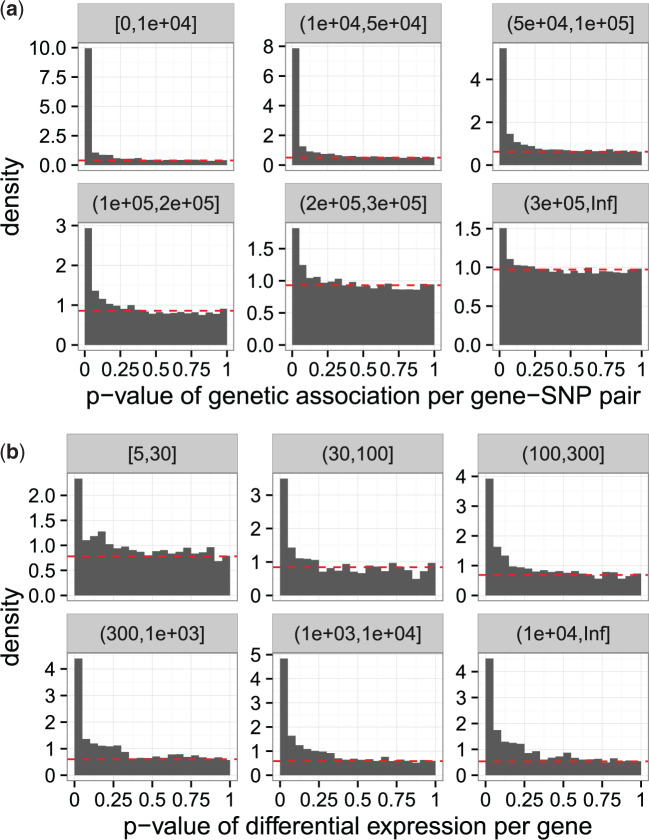
(a) *P*-value histograms of Wilcoxon tests for genetic association between genes and SNPs for the eQTL experiment in Smith and Kruglyak (2008), divided into six strata based on the gene–SNP basepair distance indicated by the strip names. The null hypothesis is “no association between a gene–SNP pair”. (b) *P*-value histograms for assessing differential gene expression in the RNA-seq study in Bottomly *and others* (2011), divided into six strata based on per-gene read depth indicated by the strip names. The null hypothesis is “no differential expression (for a gene) between two conditions”. In each subplot, the estimated proportion of true null hypotheses for all hypotheses in the corresponding stratum is based on Storey (2002) and indicated by the horizontal dashed line. It can be seen that gene–SNP genetic distance or per-gene read depth affects the prior probability of a gene–SNP association or differential gene expression.

Figure 1 shows results from multiple testing on a genetics of gene expression study (Smith and Kruglyak, 2008) and an RNA-seq differential expression study (Bottomly *and others*, 2011). In the genetics of gene expression study, the *p*-values are subdivided according to six different gene–SNP basepair distance strata. In the RNA-seq study, the *p*-values are subdivided into six different strata of per-gene read depth. It can be seen in both cases that the proportion of true null hypotheses and the power to identify significant tests vary in a systematic manner across the strata. The goal of this article is to take advantage of this phenomenon so that we may improve the accuracy of calling tests significant and do so without having to create artificial strata as in Figure 1.

We propose the *functional FDR* (fFDR) methodology that efficiently incorporates additional quantitative information for estimating the FDR and *q*-values. Specifically, we code additional information into a quantitative *informative variable* and extend the Bayesian framework for FDR pioneered in Storey (2003) to incorporate this informative variable. This leads to a functional proportion of true null hypotheses (or “functional null proportion” for short) and a functional power function. From this, we derive the optimal decision rule utilizing the informative variable. We then provide estimates of functional FDRs, functional local FDRs, and functional *q*-values to utilize in practice.

Related ideas have been developed, such as *p*-value weighting (Genovese *and others*, 2006; Roquain and van de Wiel, 2009; Hu *and others*, 2010; Ignatiadis *and others*, 2016; Ignatiadis and Huber, 2018), stratified FDR control (Sun *and others*, 2006), stratified local FDR thresholding (Ochoa *and others*, 2015), and covariate-adjusted conditional FDR estimation (Boca and Leek, 2018). Stratified FDR and local FDR rely on clearly defined strata, which may not always be available or make the best use of information. Covariate-adjusted conditional FDR estimation focuses on only one component of the FDR. *P*-value weighting has been a successful strategy. The methods in Genovese *and others* (2006) and Roquain and van de Wiel (2009) regard each hypothesis as a group and assign a weight to the *p*-value associated with a hypothesis, whereas those in Hu *and others* (2010), Ignatiadis *and others* (2016), and Ignatiadis and Huber (2018) partition hypotheses in groups and assign a weight to all *p*-values in a group. In particular, weights for the “independent hypothesis weighting (IHW)” method proposed by Ignatiadis *and others* (2016) and Ignatiadis and Huber (2018) are derived from non-trivial optimization algorithms. However, for a *p*-value weighting method, it remains challenging to derive weights that indeed result in improved power subject to a target FDR level, and how to obtain optimal weights under different optimality criteria is still an open problem. Furthermore, for a *p*-value weighting procedure that is also based on partitioning hypotheses into groups, its inferential results can be considerably affected by how the groups are formed.

Our methodology serves as an alternative to *p*-value weighting. We are motivated by similar scientific applications as the IHW method which employs a covariate to improve the power of multiple testing and is based on partitioning hypotheses into groups and *p*-value weighting. The authors of the IHW method have shown that this method has advantages over several existing methods including those of Benjamini and Hochberg (1995), Hu *and others* (2010), Scott *and others* (2015), and Cai and Sun (2009). However, our approach is distinct from the IHW method since the latter is a weighted version of the Benjamini–Hochberg procedure (Benjamini and Hochberg, 1995). In contrast, we work with a Bayesian model developed in Storey (2003), provide direct calculations of optimal significance thresholds, and take an empirical Bayes strategy to estimate several FDR quantities. As earlier frequentist and Bayesian approaches to the FDR in the standard scenario both proved to be important, our contribution here should serve to complement the *p*-value weighting strategy.

To demonstrate the effectiveness of our proposed methodology, we conduct simulations and analyze two genomics studies, an RNA-seq differential expression study and a genetics of gene expression study. In doing so, we uncover important operating characteristics of the fFDR methodology and we also provide several strategies for visualizing and interpreting results. Although our applications are focused on genomics, we anticipate the framework presented here will be useful in a wide range of scientific problems.

This rest of the article is organized as follows. We formulate the fFDR methodology in Section 2 and provide its implementation in Section 3. Two applications of the methodology are given in Section 4. We end the article with a discussion in Section 5.

## 2. The functional FDR framework

In this section, we formulate the fFDR theory and methodology. To this end, we first introduce our model, provide formulas for the positive false discovery rate (pFDR), positive false non-discovery rate (pFNR), and *q*-value, and then describe the significance rule based on the *q*-value.

### 2.1. Joint model for p-value, hypothesis status, and informative variable

Let $Z$ be the informative variable that is uniformly distributed on the interval $\left[  0,1\right]$, i.e., $Z \sim \mathsf{Uniform}\left(  0,1\right)$. For example, $Z$ can denote the quantiles of the per-gene read depths in an RNA-seq study or the quantiles of the genomic distances in an eQTL experiment. Denote the status of the null hypothesis by $H$, such that $H=0$ when the null hypothesis is true and $H=1$ when the alternative hypothesis is true. We assume that conditional on $Z=z$ the null hypothesis is *a priori* true with probability $\pi_{0}\left(  z\right)$, i.e.,

(2.1) \begin{equation*}\label{es21} (H|\ Z=z) \sim\mathsf{Bernoulli}\left( 1-\pi_{0}\left( z\right)\right), \end{equation*}

where the function $\pi_{0}(z)$ ranges in $\left[  0,1\right]  $. We call $\pi_{0}(z)$ the “prior probability of the null hypothesis”, “functional proportion of true null hypotheses”, or “functional null proportion.” When $\pi_{0}(z)$ is constant, it will be simply denoted by $\pi_0$.

To formulate the distribution of the *p*-value, $P$, we assume the following: (i) when the null hypothesis is true, $(P | H=0, Z) \sim \mathsf{Uniform}\left(  0,1\right)$ regardless of the value of $Z$; (ii) when the null hypothesis is false, the conditional density of $(P | H=1, Z=z)$ is $f_{1}\left(\cdot|z\right)$. The conditional density of $P$ given $Z=z$ is then

(2.2) \begin{equation*}\label{es20} \begin{aligned} f\left( p|z\right) &= f(p|z,H=0)\Pr(H=0|z) + f(p|z,H=1)\Pr(H=1|z) \\ &= \pi_{0}\left( z\right) +(1-\pi_{0}\left( z\right))f_{1}\left( p|z\right). \end{aligned} \end{equation*}

Since $Z$ has constant density $f(z)=1$, the joint density $f\left(  p,z\right) = f\left(  p|z\right)$ for all $(p,z) \in [0,1]^2$ so that

(2.3) \begin{equation*}\label{eqJointMd} f(p,z) = \pi_{0}\left( z\right) +(1-\pi_{0}\left( z\right))f_{1}\left( p|z\right). \end{equation*}

$Z \sim \mathsf{Uniform}\left(  0,1\right)$ and the representation in equation (2.3) are important since they enable more straightforward estimation of $f(p, z)$ than $f(p | z)$.

### 2.2. Optimal statistic

Now suppose there are $m$ hypothesis tests, with $H_i$ for $i = 1, 2, \ldots, m$ indicating the status of each hypothesis test as above. For example, $H_i$ can denote whether gene $i$ is differentially expressed or not, or whether there is an association between the $i$th gene–SNP pair. For the $i$th hypothesis test, let its calculated *p*-value be $p_i$ and its measured informative variable be $z_i$; additionally, let $P_i$ and $Z_i$ be their respective random variable representations.

Let $T=(P,Z)$ and $T_{i}=\left(P_{i},Z_{i}\right)$ for $1 \le i \le m$. Suppose the triples $(T_{i},H_i)$ are independent and identically distributed (i.i.d.) as $(T,H)$. If the same significance region $\Gamma$ in $[0,1]^2$ is used for the $m$ hypothesis tests, then identical arguments in the proof of Theorem 1 in Storey (2003) imply that $\mathrm{p}\mathrm{F}\mathrm{D}\mathrm{R}(\Gamma)=\Pr(H=0|T \in \Gamma)$ and $\mathrm{p}\mathrm{F}\mathrm{N}\mathrm{R}(\Gamma)=\Pr(H=1|T \notin \Gamma)$, where pFDR is the positive false discovery rate and pFNR is the false non-discovery rate as defined in Storey (2003). The bivariate function

(2.4) \begin{equation*}\label{es17} r\left( p,z\right) =\dfrac{\pi_{0}\left( z\right) }{f\left( p,z\right)} \end{equation*}

defined on $[0,1]^2$ is the posterior probability that the null hypothesis is true given the observed pair $(p,z)$ of *p*-value and informative variable. Note that $r(p,z)=\Pr(H=0|T=(p,z))$. So, $r(p,z)$ is an extension of the local FDR (Efron *and others*, 2001; Storey, 2003) also known as the posterior error probability (Kall *and others*, 2008). Straightforward calculation shows that

(2.5) \begin{equation*}\label{eqIntegralRep} \mathrm{p}\mathrm{F}\mathrm{D}\mathrm{R}(\Gamma) = \int_{\Gamma} r\left( p,z\right)dpdz \quad \text{and} \quad \mathrm{p}\mathrm{F}\mathrm{N}\mathrm{R}(\Gamma) = \int_{[0,1]^2 \setminus \Gamma} (1-r\left( p,z\right))dpdz \end{equation*}

Define significance regions $\{\Gamma_{\tau}:\tau \in [0,1]\}$ with

(2.6) \begin{equation*}\label{eqBayesStat} \Gamma_{\tau} = \left\{(p,z) \in [0,1]^2: r(p,z) \leq \tau\right\} \end{equation*}

such that test $i$ is statistically significant if and only if $T_i \in \Gamma_{\tau}$. Then by identical arguments in Section 6 leading up to Corollary 4 in Storey (2003), $\Gamma_{\tau}$ gives the Bayes rule for the Bayes error

(2.7) \begin{equation*}\label{eq:Bayesloss} \textrm{BE}(\Gamma_{\tau}) = (1-\tau)\Pr(T_i \in \Gamma_{\tau},H_i=0) + \tau\Pr(T_i \notin \Gamma_{\tau},H_i =1) \end{equation*}

for each $\tau \in [0,1]$. Therefore, by arguments analogous to those in Storey (2003), $r(p,z)$ is the optimal statistic for the Bayes rule with Bayes error (2.7).

### 2.3. Q-value based decision rule

With the statistic $r(p,z)$ in (2.4) and nested significance regions $\left\{\Gamma_{\tau}: \tau \in \left[0,1\right]\right\}$ with $\Gamma_{\tau}$ defined by (2.6), the definition of *q*-value in Storey (2003) implies that the *q*-value for the observed statistic $t=\left(p,z\right)$ is

(2.8) \begin{equation*}\label{eq:defffqva} q\left(p,z\right)=\inf_{\left\{\Gamma_{\tau}: t \in \Gamma_{\tau}\right\}} \mathrm{p}\mathrm{F}\mathrm{D}\mathrm{R} \left(\Gamma_{\tau}\right) =\mathrm{p}\mathrm{F}\mathrm{D}\mathrm{R} \left(\Gamma_{r(p,z)}\right)\!, \end{equation*}

where the second equality follows from Theorem 2 in Storey (2003), noting that $\{\Gamma_{\tau}\}$ are constructed from the posterior probabilities $r(p,z)$. Estimating the *q*-value in (2.8) will be discussed in Section 3.3.

Let $q(p_i,z_i)$ denote the *q*-value of $t_i=\left(p_i,z_i\right)$ for the $i$th null hypothesis $H_i$. At a target pFDR level $\alpha \in \left[ 0,1\right]$, the following significance rule

(2.9) \begin{equation*}\label{eq:fqvalueRej} ``\text{call the } i \text{th null hypothesis } H_i \text{ significant when } q(p_i,z_i) \leq \alpha\text{''} \end{equation*}

has pFDR no larger than $\alpha$. Note that this significance rule is identical to the significance regions $\{\Gamma_\tau\}$ from above, so significance rule (2.9) also achieves the Bayes optimality for the loss function (2.7). We refer to (2.9) as the “Oracle”, which will be estimated by a procedure detailed below in Section 3. When only *p*-values $\left\{p_i\right\}_{i=1}^m$ are used, the above significance rule becomes

(2.10) \begin{equation*}\label{eq:qvalueRej} ``\text{call the } i \text{th null hypothesis } H_i \text{ significant when } q(p_i) \leq \alpha\text{''}, \end{equation*}

where $q(p_i)$ is the original *q*-value for $p_i$ as developed in Storey (2002) and Storey (2003).

## 3. Implementation of the fFDR methodology

We aim to implement the decision rule in (2.9) for the fFDR methodology by a plug-in estimation procedure. For this, we need to estimate the two components of the statistic $r(p,z)$ given in (2.4): the functional null proportion $\pi_{0}(z)$ and the joint density $f(p,z)$ with support on $\left[ 0,1\right]^2$. We also need to estimate the *q*-value defined in (2.8). We will provide in Section 3.1 three complementary methods to estimate $\pi_{0}(z)$, in Section 3.2 a kernel-based method to estimate $f(p,z)$, and in Section 3.3 the estimation of *q*-values and the plug-in procedure.

### 3.1. Estimating the functional null proportion

Our proposed approaches to estimate the functional null proportion $\pi_{0}(z)$ are based on an extension of the approach taken in Storey (2002). Recalling that $\pi_{0}\left(  z\right)  =\Pr\left(  H=0|Z=z\right)$, it follows that for each $\lambda\in [0,1)$,

$$\begin{align*}
\frac{\mathrm{Pr}(P>\lambda | Z=z)}{1-\lambda} & \geq  \frac{\mathrm{Pr}(P>\lambda |H=0,Z=z) \mathrm{Pr}(H=0|Z=z)}{1-\lambda}  \\
\ &= \frac{(1-\lambda) \mathrm{Pr}(H=0|Z=z)}{1-\lambda}  \\
\ &= \pi_{0}\left(  z\right).
\end{align*}$$

If we define the indicator function $\xi_{\lambda}\left(  z\right)  =1_{\left\{  P>\lambda|Z=z\right\}  }$, then

(3.11) \begin{equation*}\label{eq:xi_est} \frac{\mathrm{E}\left[ \xi_{\lambda}\left( z\right) \right]}{1-\lambda} = \frac{\Pr(P>\lambda|Z=z)}{1-\lambda}. \end{equation*}

Therefore, $\mathrm{E}\left[  \xi_{\lambda}\left(  z\right)  \right]/(1-\lambda)$ is a conservative estimate of $\pi_{0}\left(  z\right)$ and it will form the basis of our estimate of $\pi_{0}(z)$.

Our first method to estimate $\pi_{0}(z)$ is referred to as the “GLM method” since it estimates $\mathrm{E}\left[  \xi_{\lambda}\left(  z\right)  \right] $ using generalized linear models (GLMs). For each $z\in\left[  0,1\right]  $, we let $\eta(z)=\beta_{0}+\beta_{1}z$ and

(3.12) \begin{equation*} g(z;\beta_{0},\beta_{1})=\dfrac{1-\lambda}{1+\exp\left( -\eta(z)\right) } \label{es8} \end{equation*}

for two parameters $\left(  \beta_{0},\beta_{1}\right)  $, and then fit

(3.13) \begin{equation*} \xi_{\lambda}\left( z\right) \sim\mathsf{Bernoulli}\left( g(z;\beta_{0} ,\beta_{1})\right) \label{es7} \end{equation*}

to obtain an estimate $\left(  \hat{\beta}_{0},\hat{\beta}_{1}\right)  $ of $\left(  \beta_{0},\beta_{1}\right)  $ using the paired realizations $\left\{  \left(z_{i},p_{i}\right)\right\}  _{i=1}^{m}$. We then estimate $\pi_{0}\left(z\right)$ by

(3.14) \begin{equation*}\label{es3} \hat{\pi}_{0}\left( z;\lambda\right) =\frac{g(z;\hat{\beta}_{0},\hat{\beta}_{1})}{1-\lambda}. \end{equation*}

Our second method to estimate $\pi_{0}(z)$ is referred to as the “GAM method” since it estimates $\mathrm{E}[\xi_{\lambda}\left(  z\right)]$ using generalized additive models (GAMs). Specifically, we model $\eta$ in the GLM method as a nonlinear function of $z$ via GAM, while keeping the functional form of $g$ in (3.12) and the estimator (3.14). For example, $\eta$ can be modeled by B-splines (Hastie and Tibshirani, 1986; Wahba, 1990) whose degree can be chosen, e.g., by generalized cross-validation (GCV) (Craven and Wahba, 1978). The GAM method removes the restriction induced by the GLM method that $\pi_{0}(z)$ be a monotone function of $z$.

Our third method to estimate $\pi_{0}(z)$ is referred to as the “Kernel method” since it estimates $\mathrm{E}\left[  \xi_{\lambda}\left(z\right)\right] $ via kernel density estimation (KDE). Since $Z \sim \mathsf{Uniform}\left(  0,1\right)$, it follows that

(3.15) \begin{equation*} \mathrm{E}\left[ \xi_{\lambda}\left( z\right) \right] = \Pr\left( Z=z|P>\lambda \right) \Pr\left( P>\lambda\right) \text{.}\label{es27} \end{equation*}

To estimate $\mathrm{E}\left[  \xi_{\lambda}\left(  z\right)  \right]$, we estimate the two factors in the right-hand side of (3.15) separately. It is straightforward to see that the estimator from Storey (2002),

(3.16) \begin{equation*}\label{es25} \hat{\pi}_{0}^{S}\left( \lambda\right) = \frac{\sum\nolimits_{i=1}^{m}1_{\left\{ p_{i}>\lambda\right\}} }{m(1-\lambda)} \quad \text{for} \quad\lambda\in [0,1), \end{equation*}

is a conservative estimator of $\Pr(P>\lambda)/(1-\lambda)$. Further, if we let $\hat{h}_{\lambda}$ be a conservative estimator of the density of the $z_{i}$’s whose corresponding *p*-values are greater than $\lambda$, then $\hat{h}_{\lambda}\left(  z\right)$ conservatively estimates $\Pr\left(  Z=z|P>\lambda\right)$. Correspondingly,

(3.17) \begin{equation*} \hat{\pi}_{0}\left( z;\lambda\right) =\hat{h}_{\lambda}\left( z\right) \times\hat{\pi}_{0}^{S}\left( \lambda\right)\label{es26} \end{equation*}

is a conservative estimator of $\pi_0(z)$. In the implementation, we obtain $\hat{h}_{\lambda}(z)$ using the methods in Geenens (2014) since $z$ ranges in the unit interval. Note that (3.17) is essentially a nonparametric alternative to (3.14) since $\hat{h}_{\lambda}(z)  $ does not have the constraint on its shape that $g(z;\hat{\beta}_{0},\hat{\beta}_{1})$ does.

To maintain a concise notation, we write $\hat{\pi}_{0}\left(z;\lambda\right)$ as $\hat{\pi}_{0}(z)$. If no information on the shape of $\pi_{0}(z)$ is available, we recommend using the Kernel or GAM method to estimate $\pi_{0}(z)$; if $\pi_{0}(z)$ is monotonic in $z$, then the GLM method is preferred. An approach for automatically handling the tuning parameter $\lambda$ for the estimators is provided in Section 2 of the supplementary material available at *Biostatistics* online.

### 3.2. Estimating the joint density $f(p,z)$

The estimation of the joint density $f(p,z)$ of the *p*-value $P$ and informative variable $Z$ involves two challenges: (i) $f$ is a density function defined on the compact set $[0,1]^2$; (ii) $f(p,z)$ may be monotonic in $p$ for each fixed $z$, requiring its estimate to also be monotonic. In fact, in the simulation study in Section 1 of the supplementary material available at *Biostatistics* online, $f(p,z)$ is monotonic in $p$. To deal with these challenges, we estimate $f$ in a two-step procedure as follows. Firstly, to address the challenge of density estimation on a compact set, we use a local likelihood KDE method with a probit density transformation (Geenens, 2014) to obtain an estimate $\tilde{f}(p, z)$ of $f(p, z)$, where an adaptive nearest-neighbor bandwidth is chosen via GCV. Secondly, if $f(p, z)$ is known to be monotonic in $p$ for each fixed $z$, then we utilize the algorithm in Section 3 of the supplementary material available at *Biostatistics* online to produce an estimated density $\hat{f}(p,z)$ that has the same monotonicity property as $f(p, z)$ at the observations $\{(p_i,z_i)\}_{i=1}^m$.

### 3.3. FDR and q-value estimation

With the estimates $\hat{\pi}_{0}(z;\lambda)$ and $\hat{f}(p,z)$, respectively for $\pi_{0}(z)$ and $f(p,z)$, the functional posterior error probability (or local FDR) statistic $r(p,z)$ in (2.4) is estimated by

(3.18) \begin{equation*}\label{eq:lfdrhat} \hat{r}\left( p,z\right) =\dfrac{\hat{\pi}_{0}\left( z\right) }{ \hat{f}(p, z) }. \end{equation*}

For a threshold $\tau$, Storey *and others* (2005) proposed the following pFDR estimate

$$ \widehat{\mbox{pFDR}}\left(\Gamma_\tau\right)= \dfrac{1}{| \mathcal{S}_\tau|}\sum_{j \in \mathcal{S}_\tau}\hat{r}(p_j,z_j), \ \ \text{where} \  \mathcal{S}_\tau=\left\{ j :\hat{r}(p_j,z_j)\leq \tau\right\}.$$

The rationale for this estimate is that the numerator is the expected number of false positives given the posterior distribution $\hat{r}(p,z)$ and the denominator is the expected number of total discoveries given $\hat{r}(p,z)$ (which is directly observed). This is related to a semiparametric Bayesian procedure detailed in Newton *and others* (2004). Given this, the functional *q*-value $q(p_i,z_i)$ of $t_i=(p_i,z_i)$ corresponding to the $i$th null hypothesis $H_i$ is estimated by:

(3.19) \begin{equation*}\label{eq:estQvalue2d} \hat{q}(p_i,z_i)=\dfrac{1}{| \mathcal{S}_i|}\sum_{j \in \mathcal{S}_i}\hat{r}(p_j,z_j), \ \ \text{where} \ \mathcal{S}_i=\left \{ j :\hat{r}(p_j,z_j)\leq\hat{ r}(p_i,z_i) \right \}. \end{equation*}

The plug-in decision rule is to call the null hypothesis $H_i$ significant whenever $\hat{q}(p_i,z_i) \leq \alpha$ at a target pFDR level $\alpha$. In this work, we refer to this rule as the “functional FDR (fFDR) method”.

Recall that an estimate of $\hat{q}(p_i)$ of the *q*-value $q(p_i)$ for $p_i$ can be obtained by the *q*-value package (Storey *and others*, 2019). Then the plug-in decision rule based on $\hat{q}(p_i)$ is to call the null hypothesis $H_i$ significant whenever $\hat{q}(p_i) \leq \alpha$. This rule is referred to as the “standard FDR method” in this work. In Section 1 of the supplementary material available online at *Biostatistics*, we carry out a simulation study to demonstrate the accuracy of our estimator and compare its power to existing methods.

## 4. Applications in genomics

In this section, we apply the fFDR method to analyze data from two studies, one in a genetics of gene expression (eQTL) study on baker’s yeast and the other in an RNA-seq differential expression analysis on two inbred mouse strains. We will provide a brief background on the studies and then present the analysis results for both data sets.

### 4.1. Background on the eQTL experiment

The experiment on baker’s yeast (*Sacchromyces cerevisiae*) has been performed by Smith and Kruglyak (2008), where genome-wide gene expression was measured in each of the 109 genotyped strains under two conditions, glucose and ethanol. Here, we aim to identify genetic associations (technically, genetic linkage in this case) between pairings of expressed genes and SNPs among the samples grown on glucose. In this setting, the null hypothesis is “no association between a gene-SNP pair”, and the functional null proportion denotes the prior probability that the null hypothesis is true. The data set from this experiment is referred to as the “eQTL dataset”.

To keep the application straightforward, we consider only intra-chromosomal pairs, for which the genomic distances can be defined and are quantile normalized to give the informative variable $Z$. (Note that the fFDR framework can be used to consider all gene–SNP pairs by estimating a standard $r(p) = \pi_0/f(p)$ for all inter-chromosomal pairs and then combining these with the estimated $r(p, z)$ from the intra-chromosomal pairs to form the significance regions given in (2.6).) In this application, the genomic distance is calculated as the nucleotide basepair distance between the center of the gene and the SNP, the Wilcoxon test of association between gene expression and the allele at each SNP is used, and the *p*-values of such tests are obtained. We emphasize that the difference in the *p*-value histograms for the six strata shown in Figure 1a shows that a functional null proportion $\pi_0(z)$ is more appropriate.

### 4.2. Background on the RNA-seq study

A common goal in gene expression studies is to identify genes that are differentially expressed across varying biological conditions. In RNA-seq based differential expression studies, this goal is to detect genes that are differentially expressed based on counts of reads mapped to each gene. The null hypothesis is “no differential expression (for a gene) between the two conditions”, and the functional null proportion denotes the prior probability that the null hypothesis is true. For an RNA-seq study, the quantile normalized per-gene read depth is the informative variable $Z$ that we utilized, which affects the power of the involved test statistics (Tarazona *and others*, 2011) or the prior probability of differential expression (Robinson *and others*, 2015).

We utilized the RNA-seq data studied in Bottomly *and others* (2011), due to its availability in the ReCount database (Frazee *and others*, 2011) and because it had previously been examined in a comparison of differential expression methods (Soneson and Delorenzi, 2013). The data set, referred to as the “RNA-seq dataset”, contains 102.98 million mapped RNA-seq reads in 21 individuals from two inbred mouse strains. As proposed in Law *and others* (2014), we normalized the data using the voom R package, fitted a weighted linear least squares model to each gene expression variable, and then obtained a *p*-value for each gene based on a *t*-test of the coefficient corresponding to mouse strain.

### 4.3. Estimating the functional null proportion in the two studies

**Fig. 2.**
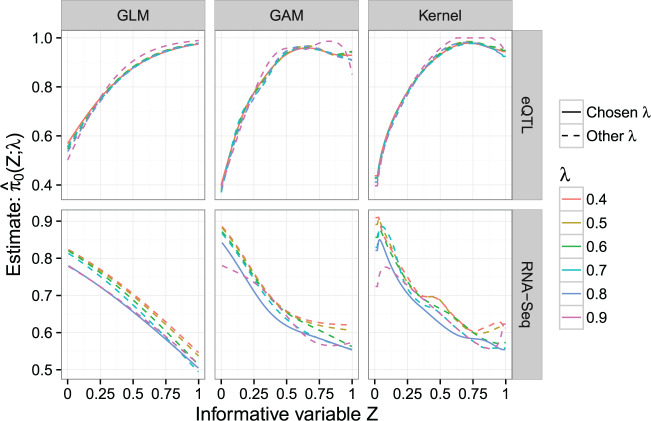
Estimate $\hat{\pi}_{0}(z;\lambda)$ of the functional null proportion $\pi_{0}(z)$ for the eQTL and RNA-seq studies, using the GLM, GAM, or Kernel method. Each plot shows the estimate $\hat{\pi}_0(z;\lambda)$ for different values of the tuning parameter $\lambda$, where the solid curve corresponds to the chosen tuning parameter value. The tuning parameter is chosen to balance the trade-off between the integrated bias and variance of the function $\hat{\pi}_{0}(z;\lambda)$; details on how to choose $\lambda$ are given in Section 2 of the supplementary material available at *Biostatistics* online.

We applied to these two data sets our estimator $\hat{\pi}_{0}(z;\lambda)$ of the functional null proportion $\pi_{0}(z)$ utilizing the GLM, GAM, and Kernel methods. Figure 2 shows $\hat{\pi}_{0}(z;\lambda)$ for these two data sets, and the tuning parameter $\lambda$ has been chosen to be the one that minimizes the mean integrated squared error of the function $\hat{\pi}_{0}(z;\lambda)$; see Section 2 of the supplementary material available at *Biostatistics* online for details on choosing $\lambda$. In both data sets, $\hat{\pi}_{0}(z;\lambda)$ based on the GAM and Kernel methods give very similar estimates, with the one based on the GLM method more distinct, likely because the latter puts stricter constraints on the shape of $\pi_{0}(z)$. By comparing Figure 2 to the results in Figure 4 (for estimating a constant $\pi_0$) of the supplementary material available at *Biostatistics* online, we see that in this RNA-seq study the read depths appear to affect the prior probability of differential expression, $\pi_{0}(z)$.

As expected, the estimator $\hat{\pi}_{0}(z;\lambda)$ for the eQTL data set increases with genomic distance, indicating that a distal gene–SNP association is less likely than local association. Using the GAM and Kernel methods, $\hat{\pi}_{0}(z;\lambda)$ ranges from about $0.54$ for very local associations to about $0.82$ for distant gene–SNP pairs. In the RNA-seq data set, $\hat{\pi}_{0}(z;\lambda)$ obtained by the GAM and Kernel methods decreases from around $0.97$ to around $0.47$ as read depth increases.

In the eQTL data set, $\lambda$ values from $0.4$ to $0.8$ led to very similar shapes of $\hat{\pi}_{0}(z;\lambda)$ (see Figure 2), and the integrated bias of $\hat{\pi}_0(z;\lambda)$ is always low compared with its integrated variance, leading to the choice of $\lambda=0.4$ for all three methods. This likely indicates that the test statistics implemented in this experiment have high power, leading to low bias in estimating $\pi_{0}(z)$. In contrast, in the RNA-seq data set, the integrated bias of $\hat{\pi}_0(z;\lambda)$ does decrease as $\lambda$ increases, leading to a choice of a higher $\lambda$. While the choice of $\lambda$ for $\hat{\pi}_{0}(z;\lambda)$ may deserve further study, it is clear from these applications that the choice of $\lambda$ has a small effect on $\hat{\pi}_{0}(z;\lambda)$ and that it is beneficial to employ a functional $\pi_{0}(z)$ of the informative variable rather than a constant $\pi_0$.

### 4.4. Application of fFDR method in the two studies

**Fig. 3.**
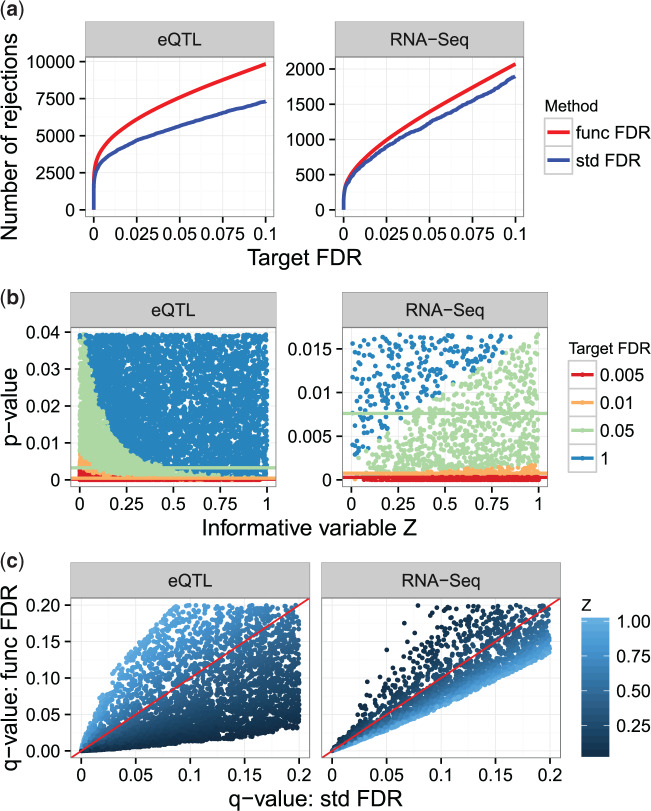
The fFDR method applied for multiple testing in the eQTL and RNA-seq analyses. (a) Number of significant hypothesis tests at various target FDRs. The fFDR method (func FDR) has more significant tests than the standard FDR method (std FDR) at all target FDRs. (b) The significance regions of the fFDR method for various target FDRs, indicated by scatter plots of the *p*-values and informative variable. The horizontal lines indicate the significance thresholds that would be used by the standard FDR method at the same target FDRs. Clearly, these lines do not take the informative variable into account. (c) A scatter plot comparing the *q*-values for the standard FDR method ($x$ axis) to the *q*-values for the fFDR method ($y$ axis), colored based on the informative variable $Z$ with reference line $x=y$ in red. It is clear that the fFDR method re-ranks the significance of hypotheses tests.

For the eQTL analysis described in Section 4.1, $\hat{\pi}_{0}(z;\lambda)$ based on the GAM method is used (see Figure 2), and the fFDR method is applied to the *p*-values of the tests of associations and the quantile normalized genomic distances. At the target FDR of $0.05$, the fFDR method found $7579$ associated gene–SNP pairs, the standard FDR method $5655$, and the two methods shared $5450$ discoveries. Figure 3a shows that, at all target FDR levels, the fFDR method has higher power than the standard FDR method. In addition, Figure 3b reveals that the significance region of the fFDR method is greatly influenced by the gene–SNP distance, as the *p*-value cutoff for significance is higher for close gene–SNP pairs and lower for distant gene–SNP pairs. This, together with Figure 3c, means that some *q*-values $q(p_i,z_i)$ for the fFDR method can be larger than the *q*-values $q(p_i)$ of the standard FDR method. Thus, the use of an informative variable by the fFDR method changes the significance ranking of the null hypotheses and increases the power of multiple testing at the same target FDR level.

For the RNA-seq analysis, the estimator $\hat{\pi}_{0}(z;\lambda)$ based on the GAM method is used (see Figure 2), and the fFDR method is applied to the *p*-values of the tests for differential expression and the quantiles of the read depths. Similar to the eQTL analysis, the fFDR method has a larger number of significant hypothesis tests at all target FDR levels; see Figure 3a. At the target FDR of $0.05$, the fFDR method found $1392$ genes to be differentially expressed, while the standard FDR method found $1231$, and the two methods shared $1202$ discoveries. In this RNA-seq analysis, the fFDR method has a smaller improvement in power (see Figure 3a), the differences between the *q*-values for the fFDR method and those for the standard FDR method are smaller (see Figure 3c), and the significance region of the fFDR method is less affected by the informative variable $Z$ (see Figure 3b). This may be because the total number of differentially expressed genes in the RNA-seq study was small or the test statistics applied in this experiment were already powerful.

## 5. Discussion

We have proposed the fFDR methodology to utilize additional information on the prior probability of a null hypothesis being true or the power of the family of test statistics in multiple testing. It employs a functional null proportion of true null hypotheses and a joint density for the *p*-values and informative variable. Our simulation studies have demonstrated that the fFDR methodology is more accurate and informative than the standard FDR method, that the former does not perform worse than the latter when the informative variable is in fact non-informative, and that the fFDR methodology is more powerful than the IHW method. (see Section 1 of the supplementary material available at *Biostatistics* online).

Besides the eQTL and RNA-seq analyses demonstrated here, the fFDR methodology is applicable to multiple testing in other studies. For example, it can be applied to genome-wide association studies such as those conducted in Dalmasso *and others* (2008) and Roeder *and others* (2006), where an informative variable can incorporate differing minor allele frequencies or information on the prior probability of a gene–SNP association obtained from previous genome linkage scans. It can also be used in brain imaging studies, e.g., those conducted or reviewed in Benjamini and Heller (2007) and Chumbley and Friston (2009), to integrate as the informative variable spatial-temporal information on the voxel measurements.

We recommend using domain knowledge to determine and choose an informative variable. In essence, any random variable that does not affect the null distribution of *p*-value is a legitimate candidate for an informative variable. On the other hand, when the informative variable is actually non-informative on the prior of a null hypothesis being true or the power of an individual test, the fFDR method reduces to the standard FDR method, and there is no loss of power employing the fFDR method (compared with the standard FDR method). It would also be useful to develop a formal statistical test to check if a random variable is informative.

Finally, the fFDR methodology can be extended to the case where *p*-values or the status of null hypotheses are dependent on each other. In this setting, the corresponding decision rule may be different from that obtained here. On the other hand, the methodology can be extended to incorporate a vector of informative variables. This could be especially appropriate when additional information cannot be compressed into a univariate informative variable. Briefly, let $\tilde{\mathbf{Z}}$ be a $d$-dimensional random vector. We can transform $\tilde{\mathbf{Z}}$ into $\mathbf{Z}$ such that $\mathbf{Z}$ is approximately uniformly distributed on the $d$-dimensional unit cube $[0,1]^d$. Assume $\mathbf{Z} \sim \mathsf{Uniform}\left( [0,1]^d\right) $ and maintain the notation for *p*-value and status of a hypothesis used in Section 2. Then the extended model has the following components: (i) $(H|\ \mathbf{Z}=\mathbf{z})  \sim\mathsf{Bernoulli}\left(  1-\pi_{0}\left(  \mathbf{z}\right)\right)$, where the function $\pi_0(\mathbf{z})$ ranges in $\left[  0,1\right]  $; (ii) when the null hypothesis is true, $(P | H=0, \mathbf{Z}) \sim$ Uniform(0,1) regardless of the value of $\mathbf{Z}$; (iii) when the null hypothesis is false, the conditional density of $(P | H=1, \mathbf{Z}=\mathbf{z})$ is $f_{1}\left(\cdot|\mathbf{z}\right)$. Consequently, the conditional density of $P$ given $\mathbf{Z}=\mathbf{z}$ is $f\left(  p|\mathbf{z}\right)= \pi_{0}\left(  \mathbf{z}\right)  +(1-\pi_{0}\left(  \mathbf{z}\right))f_{1}\left(  p|\mathbf{z}\right)$, and the joint density $f\left(  p,\mathbf{z}\right) = f\left(  p|\mathbf{z}\right)$ for all $(p,\mathbf{z}) \in [0,1]^{(1+d)}$. The estimation procedures and significance rule we have proposed can be extended accordingly.

## 6. Software

The methods described in this article are available in the fFDR R package, available at https://github.com/StoreyLab/fFDR (most recent version), which will also be made available on CRAN.

## Supplementary Material

kxz010_Supplementary_Data
