# Supplementary material for: The functional false discovery rate with applications to genomics
Source: Biostatistics. 2019 May 28;22(1):68–81. doi: 10.1093/biostatistics/kxz010 (PMC7846131; doi:10.1093/biostatistics/kxz010)
Supplement: kxz010_Supplementary_Data [file kxz010_supplementary_data.pdf]

Biostatistics (2019), **0**, 0, *pp.* 1–20
doi:10.1093/biostatistics/functional˙FDR˙supp

The Functional False Discovery Rate with Applications to Genomics “Supplementary Materials”

Xiongzhi Chen[†,1], David G. Robinson[†,2] and John D. Storey[†,*]
[†] *Lewis-Sigler Institute for Integrative Genomics, Princeton University, Princeton, NJ 08544, USA.*
[1] *Current address: Department of Mathematics and Statistics, Washington State University, Pullman, WA 99164, USA.*

[2] *Current address: DataCamp, Empire State Building, 350 5th Ave, Floor 77, New York, NY 10118, USA*

jstorey@princeton.edu

Summary

We provide a simulation study on the proposed estimation procedures and the fFDR methodology, and show how to choose the tuning parameter λ for the estimator $\hat{\pi}_0(z;\lambda)$ of the functional null proportion $\pi_0(z)$ and how to ensure that the estimator $\hat{f}(p,z)$ of the joint density $f(p,z)$ of the p-value and informative variable (P,Z) has the same monotonicity property of f when evaluated at the observations $\{(p_i,z_i)\}_{i=1}^m$.

Key words: FDR, kernel density estimation, multiple hypothesis testing.

1. Simulation study

We present a simulation study on the performance of the fFDR method. To assess the overall error rate of a multiple testing procedure (MTP), we compare the estimated q-values to the oracle

© The Author 2019. Published by Oxford University Press. All rights reserved. For permissions, please e-mail: journals.permissions@oup.com

q-values. To assess the power of an MTP, we use the expected ratio of the number of significant true alternative hypothesis tests to the total number of true alternative tests, which is the average power across all true alternative tests. Note that the "oracle q-value" and "average power" are calculated as Monte Carlo averages over the simulated data sets for each scenario based on the fact that we know the true status of each hypothesis test.

1.1 *Simulation design*

To investigate the performance of the fFDR method when the functional null proportion $\pi_0(z)$ takes different forms, we construct four types of $\pi_0(z)$, where we calculate the average value as $\tilde{\pi}_0 = \int_0^1 \pi_0(z)\,dz$:

i) *decreasing*: $\pi_0(z) = 1 - (0.9 \times z^{3.5} + 0.01)$ such that $\tilde{\pi}_0 \approx 0.7967$.

ii) *increasing*: $\pi_0(z) = 0.9 \times (z^{0.3} + 0.1)$ such that $\tilde{\pi}_0 \approx 0.7784$.

iii) *constant*: $\pi_0(z) = \pi_0 = 0.85$ for all values of z.

iv) *sine*: $\pi_0(z) = 0.2 + 0.78 \times \sin(\pi z)$ such that $\tilde{\pi}_0 \approx 0.6992$.

To investigate how the performance of the fFDR method is affected by the informative variable Z through the conditional p-value density $f_1(p|z)$ under the false null hypothesis, we construct two types of $f_1(p|z)$:

i) *dependent*: $f_1(p|z) \propto p^{\alpha_0 - 1}(1-p)^{\beta_0 - 1}$, where $\alpha_0 = 0.3$, $\gamma_0 = 4.5$, $\beta_0 = \gamma_0 + 1.4z$, where the scalar 1.4 helps reduce the chance of generating large p-values under the false null hypothesis.

ii) *independent*: $f_1(p|z) \propto p^{\alpha_0 - 1}(1-p)^{\gamma_0 - 1}$ with $\alpha_0 = 0.3$, $\gamma_0 = 4.5$, where $f_1(p|z)$ is independent from z.

The choices of $\pi_0(z)$ are based on our observations from real data sets that $\pi_0(z)$ infrequently assumes the value 0 or 1, and that the mean of $\pi_0(Z)$ is not very small. Note that $f_1(p|z)$ for each fixed z is from the Beta density family. With the chosen α_0 and β_0, for each fixed z the p-value under the false null hypothesis has a decreasing density, is stochastically smaller than the $\mathsf{Uniform}(0,1)$ random variable, and has positive probability to assume a range of small values. This enables the competing MTPs (described in the next paragraph) to have reasonable power. Further, the densities $f_1(\cdot|z)$ indexed by z for the p-values under the false null hypothesis tend to satisfy $f_1(p|z) \leqslant f_1(p|z')$ for large p when $z \leqslant z'$, and therefore z indexes the power of the test statistic that produces the corresponding p-value. The characteristics of $\pi_0(z)$ and $f_1(p|z)$ are intended to make the simulation study both practical and fair to the competing MTPs.

For each of the eight configurations given above (four versions of $\pi_0(z)$ by two versions of $f_1(p|z)$), we simulated 100 data sets for $m = 3000$ tests according to the following procedure:

1. Generate $\{z_i\}_{i=1}^m$ independently from $\mathsf{Uniform}(0,1)$.

2. For each $i = 1, 2, \ldots, m$, generate $H_i|z_i \sim \mathsf{Bernoulli}(1 - \pi_0(z_i))$; if $H_i = 0$, generate p_i from $\mathsf{Uniform}(0,1)$ and if $H_i = 1$ generate p_i according to density $f_1(p|z_i)$.

3. Apply the standard FDR method, the fFDR method, the Oracle and the IHW method with q-value cut-offs at $0.01, 0.02, \ldots, 0.1$. The IHW method is implemented by the R package IHW via its default setting. Calculate the true FDR and average power for each MTP. The quantities $\pi_0(z)$, $f_1(p|z)$, $f(p,z)$, $r(p,z)$ and $q(p,z)$ are all estimated using $\tilde{z}_i = \tilde{r}_i/m$ for $i = 1, ..., m$, where $\tilde{r}_i$ is the rank of z_i among $\{z_j\}_{j=1}^m$ and z_i is referred to as the "quantile transformed" value of $\tilde{z}_i$.

To assess the performance of the fFDR method when the assumption $Z \sim \mathsf{Uniform}(0,1)$ is violated but all other settings given above are the same, we simulate m $\tilde{z}_i$'s independently from $\mathsf{NegBinomial}(5, 0.65)$, i.e., the Negative Binomial distribution with 5 successful trials for which

each trial has probability of success 0.65, or from the "Uniform-Negative-Binomial" mixture as $0.5 \times \mathsf{Uniform}\,(0,1) + 0.5 \times \mathsf{NegBinomial}(5, 0.65)$. Each $\tilde{z}_i$ is then quantile transformed into $z_i = \tilde{r}_i/m$. It is easy to check that z_i's based on $\mathsf{Uniform}\,(0,1)$ or the Uniform-Negative-Binomial mixture are approximately uniformly distributed and that z_i's based on the Negative Binomial distribution are far from being uniformly distributed.

1.2 *Simulation results*

We first examined the power and the estimation accuracy for the four methods, where the fFDR method was applied using the GAM model to estimate $\pi_0(z)$. Figure 1 displays the average power with respect to the applied q-value cut-offs. The following can be seen: (i) the power of the fFDR method is very close to that of the Oracle; (ii) the power of the fFDR method is greater than that of the standard FDR method when the functional null proportion $\pi_0(z)$ is not constant; (iii) the power of the fFDR method is no smaller than that of the standard FDR method when $\pi_0(z)$ is constant; (iv) the fFDR method is (much) more powerful than the IHW method when $\pi_0(z)$ is not constant, and the IHW method is more powerful than the standard FDR method when $\pi_0(z)$ is monotone; (v) the IHW method has almost identical power as the standard FDR method when $\pi_0(z)$ is constant or the sine function. It should be noted that, when $\pi_0(z)$ is not constant, the fFDR method achieves ∼10% to 30% increase in power over the standard FDR method.

Figure 2 shows the estimated q-values versus the oracle q-values, where the oracle q-values are computed using the optimal statistic given in (2.4) of the main text. It shows that the fFDR method estimates the oracle q-values accurately and that it does so more accurately than the standard FDR method. In summary, the fFDR method in these simulations is more powerful than the standard FDR method when the functional null proportion is nonconstant and it estimates the FDR accurately. Figure 3 shows the performance of the estimator $\hat{\pi}_0(z;\lambda)$ implemented by the GAM, GLM, and Kernel methods. We see that these estimates are accurate but may be

slightly downwardly biased in subsets of the range of $\pi_0(z)$. One exception is the GLM estimate in the *sine* scenario, but this is expected given that the GLM model has only a linear term in z and is not flexible enough to capture the sine shape. The GLM model could of course be modified to include higher order polynomial terms in z to make it more flexible.

Figure 1 shows that when π_0 is a constant, there is not an increase in power of the fFDR method over the standard FDR method. We investigated why this might be the case. We first examined the accuracy of estimating π_0 when it is a constant, shown in Figure 4. The GAM and Kernel methods yield similar estimates, whereas the GLM method is almost a constant and very close to the estimate given by the `qvalue` package (Storey et al., 2015). Whereas the GLM, GAM, and Kernel methods yield similar estimates in the *decreasing* and *increasing* scenarios of $\pi_0(z)$ (Figure 3) as well as in the real data analysis shown in Figure 2 of the main text, one can see that the GLM method may be more accurate in the case that π_0 is constant. We therefore repeated the simulations for the *constant* scenario where the true π_0 was substituted for the estimated $\pi_0(z)$ in the fFDR method and for the estimated π_0 in the standard FDR method. The power comparisons between fFDR and the standard FDR were qualitatively similar to those observed in the third row of Figure 1.

This then focused our attention on the estimation of the $f(p, z)$ component from our statistic $r(p, z) = \pi_0(z)/f(p, z)$ of equation (2.4) of the main text. Besides the Beta family used in defining $f(p, z)$ in the simulations, we defined $f(p, z)$ from several other distributions (including those induced by Normal and t distributions), but we continued to see similar levels of power between the fFDR method and the standard FDR. An explanation for this is that, when the signal strengths are moderate or strong, the p-values already capture much of the power characteristics of the test statistics so that $f(p, z) \approx f(p)$. In this case, the informative variable may not be influential in the $f(p, z)$ component of the $r(p, z)$ statistic. These findings have several relevant implications: (i) if the researcher is certain that $\pi_0(z)$ is constant, then the fFDR method can be

implemented with the estimate of a constant π_0; (ii) if the signal strengths are not weak and the fFDR method performs very similarly to the standard FDR method, then it is likely that $\pi_0(z)$ does not depend on the informative variable; (iii) it will be useful to better understand $f(p, z)$ versus $f(p)$; and (iv) it will be useful to derive a test of whether $\pi_0(z)$ is functional or constant.

The simulation results when z_i's are quantile transformed values of $\tilde{z}_i$'s that are independent and follow the Uniform-Negative-Binomial mixture (or the Negative Binomial distribution) are visualized in Figure 6, 7 and 8 (or Figure 9, 10 and 11). To save space, the functional proportion $\pi_0(z)$ is estimated by the GAM method. We have observed the following: (i) when $\tilde{z}_i$'s are independent and follow the Uniform-Negative-Binomial mixture such that z_i's are approximately uniformly distributed, the performances of the fFDR method and our procedures to estimate the fFDR model are very similar to that when z_i's are independent and follow $\mathsf{Uniform}(0, 1)$. In other words, our method and procedures are robust to how the random variable that induces the informative variable is distributed as long as the latter is approximately uniformly distributed; (ii) when z_i's are far from being approximately uniformly distributed as those based the Negative Binomial distribution, the GAM method still well estimates $\pi_0(z)$, the fFDR method is still more powerful than the standard FDR method and the IHW method when $\pi_0(z)$ is not constant, and the three methods have almost identical powers when $\pi_0(z)$ is constant. However, due serious violation of the uniformity assumption on the informative variable Z in this scenario, the power of the fFDR method may not be very close to that of the Oracle, and the fFDR method can be anti-conservative when the q-value cut-off is large and tend to over-estimate the oracle q-value when the latter is large.

2. Choice of tuning parameter for estimators of the functional null proportion

The GLM, GAM and Kernel methods of estimating the functional null proportion $\pi_0(z)$ all rely on a tuning parameter λ that controls the trade-off between the bias and variance of the estimator

$\hat{\pi}_0(z;\lambda)$. One observation we have made from the simulation studies is the following: $\hat{\pi}_0(z;\lambda)$ with smaller λ tends to capture the correct shape of $\pi_0(z)$ but with different levels of bias, while $\hat{\pi}_0(z;\lambda)$ with larger λ may have poorly estimated the shape of $\pi_0(z)$ but with less bias, all with respect to the mean

$$\tilde{\pi}_0 = \mathrm{E}\left[\pi_0\left(Z\right)\right] = \int_0^1 \pi_0(z)dz.$$

So, a simple rule of thumb is to pick a $\hat{\lambda}$ for which $\hat{\pi}(z;\hat{\lambda})$ is the least biased but still has a similar shape to those of $\hat{\pi}(z;\lambda)$ obtained with larger λ. In other words, the desired $\hat{\lambda}$ should balance the integrated bias and variance of $\hat{\pi}(z;\lambda)$. The following formalizes this intuition.

For a function φ_λ that satisfies the constraint

$$\int_0^1 \hat{\pi}_0(z;\lambda)dz = \int_0^1 \varphi_\lambda(z)dz, \tag{2.1}$$

let

$$\tilde{\omega}(\lambda) = \int_0^1 \left[\hat{\pi}_0(z;\lambda) - \varphi_\lambda(z)\right]^2 dz \tag{2.2}$$

be an approximate integrated variance of $\hat{\pi}_0(z;\lambda)$. Further, let

$$\tilde{\delta}(\lambda) = \int_0^1 \varphi_\lambda(z)dz - \int_0^1 \pi_0(z)dz \tag{2.3}$$

be an approximate integrated bias of $\hat{\pi}_0(z;\lambda)$. Set $\widetilde{\mathrm{MISE}}\left(\lambda\right) = \tilde{\omega}(\lambda) + \tilde{\delta}^2(\lambda)$. Then $\widetilde{\mathrm{MISE}}\left(\lambda\right)$ measures the "bias" and "variance" of $\hat{\pi}_0(z;\lambda)$ with respect to a reference estimate φ_λ, and serves as an estimate of the mean integrated squared error of $\hat{\pi}_0(z;\lambda)$. So, a $\hat{\lambda}$ that minimizes $\widetilde{\mathrm{MISE}}(\lambda)$ will achieve the desired trade-off without much computational cost or much loss in the accuracy in estimating $\pi_0(z)$.

With the above preparation, the procedure to determine $\hat{\lambda}$ is as follows:

1. Let $\mathcal{R} = \{.4, .5, \ldots, .9\}$ be a finite set of λ values in $(0,1)$. For each $\lambda \in \mathcal{R}$, obtain $\hat{\pi}_0(z;\lambda)$ using a method provided in Section 3.1 of the main text.

2. Let $\dot{\lambda} = \min\{\lambda : \lambda \in \mathcal{R}\}$. For each $\lambda \in \mathcal{R}$, estimate the function φ_λ as

$$\hat{\varphi}_\lambda(z) = \hat{\pi}_0(z; \dot{\lambda}) - k_\lambda \left[1 - \hat{\pi}_0(z; \dot{\lambda})\right], \tag{2.4}$$

where k_λ is chosen such that $\int_0^1 \hat{\varphi}_\lambda(z)dz = \int_0^1 \hat{\pi}_0(z; \lambda)dz$. Use $\hat{\varphi}_\lambda$ to estimate $\tilde{\omega}(\lambda)$ as

$$\omega(\lambda) = \int_0^1 \left[\hat{\pi}_0(z; \lambda) - \hat{\varphi}_\lambda(z)\right]^2 dz. \tag{2.5}$$

3. Use the method in Storey et al. (2004) to compute $\hat{\pi}_0^S$ in (3.16) of the main text as an estimate of the mean $\tilde{\pi}_0 = \mathrm{E}\left[\pi_0(Z)\right]$; use $\hat{\pi}_0^S$ to estimate $\tilde{\delta}(\lambda)$ as

$$\delta(\lambda) = \int_0^1 \hat{\pi}_0(z; \lambda)dz - \hat{\pi}_0^S. \tag{2.6}$$

4. Set

$$\mathrm{MISE}(\lambda) = \omega(\lambda) + \delta^2(\lambda) \tag{2.7}$$

as an estimate of $\widetilde{\mathrm{MISE}}(\lambda)$, and choose $\hat{\lambda}$ as

$$\hat{\lambda} = \mathrm{argmin}_{\lambda' \in \mathcal{R}} \mathrm{MISE}(\lambda') = \mathrm{argmin}_{\lambda' \in \mathcal{R}} \left\{\omega(\lambda') + \delta^2(\lambda')\right\}. \tag{2.8}$$

Set $\hat{\pi}_0(z; \hat{\lambda})$ as the estimate of $\pi_0(z)$.

Figure 5 illustrates how λ is chosen when estimating $\pi_0(z)$ for each of the data sets analyzed in Section 5.3 of the main text.

3. Ensuring a monotonicity property of the estimator of the joint density

Recall that the joint density for the p-value and informative variable (P, Z) satisfies

$$f(p, z) = \pi_0(z) + (1 - \pi_0(z)) f_1(p|z),$$

where $f_1(p|z)$ is the conditional density of the p-value under the true alternative hypothesis given $Z = z$. If $f_1(p|z)$ is non-increasing in p for each fixed z, then so is $f(p, z)$ and so should be its

estimate $\hat{f}(p,z)$. In order to make $\hat{f}(p,z)$ have such a monotonicity property when evaluated at the observations $\{(p_i,z_i), 1 \leqslant i \leqslant m\}$, we modify the estimate $\tilde{f}(p,z)$ of $f(p,z)$ discussed in Section 3.2 of the main text as follows. For each $i = 1,\dots,m$, set

$$\hat{f}(p_i,z_i) = \min\left\{\tilde{f}(p_i,z_i), \min\left\{\tilde{f}(p_j,z_j) : p_j < p_i \text{ and } |z_i - z_j| \leqslant \epsilon\right\}\right\},$$

where ϵ (by default being 0.02 in this paper) is chosen to be small and positive because $Z \sim \mathsf{Uniform}(0,1)$ and $\Pr(z_i = z_j) = 0$ for $i \neq j$. This guarantees that, for all pairs $(p_i,z_i),(p_j,z_j) \in [0,1]^2$,

$$p_i \geqslant p_j \text{ and } |z_i - z_j| \leqslant \epsilon \implies \hat{f}(p_i,z_i) \leqslant \hat{f}(p_j,z_j).$$

In the unusual event that $f(p,z)$ is non-decreasing in p for each fixed z, then an analogous algorithm can be performed satisfying the non-decreasing property.

References

Storey, J. D., Bass, A. J., Dabney, A. R., and Robinson, D. (2015). *qvalue: Q-value estimation for false discovery rate control.* R package version 2.1.1.

Storey, J. D., Taylor, J. E., and Siegmund, D. (2004). Strong control, conservative point estimation in simultaneous conservative consistency of false discover rates: a unified approach. *Journal of the Royal Statistical Society, Series B*, 66(1):187–205.

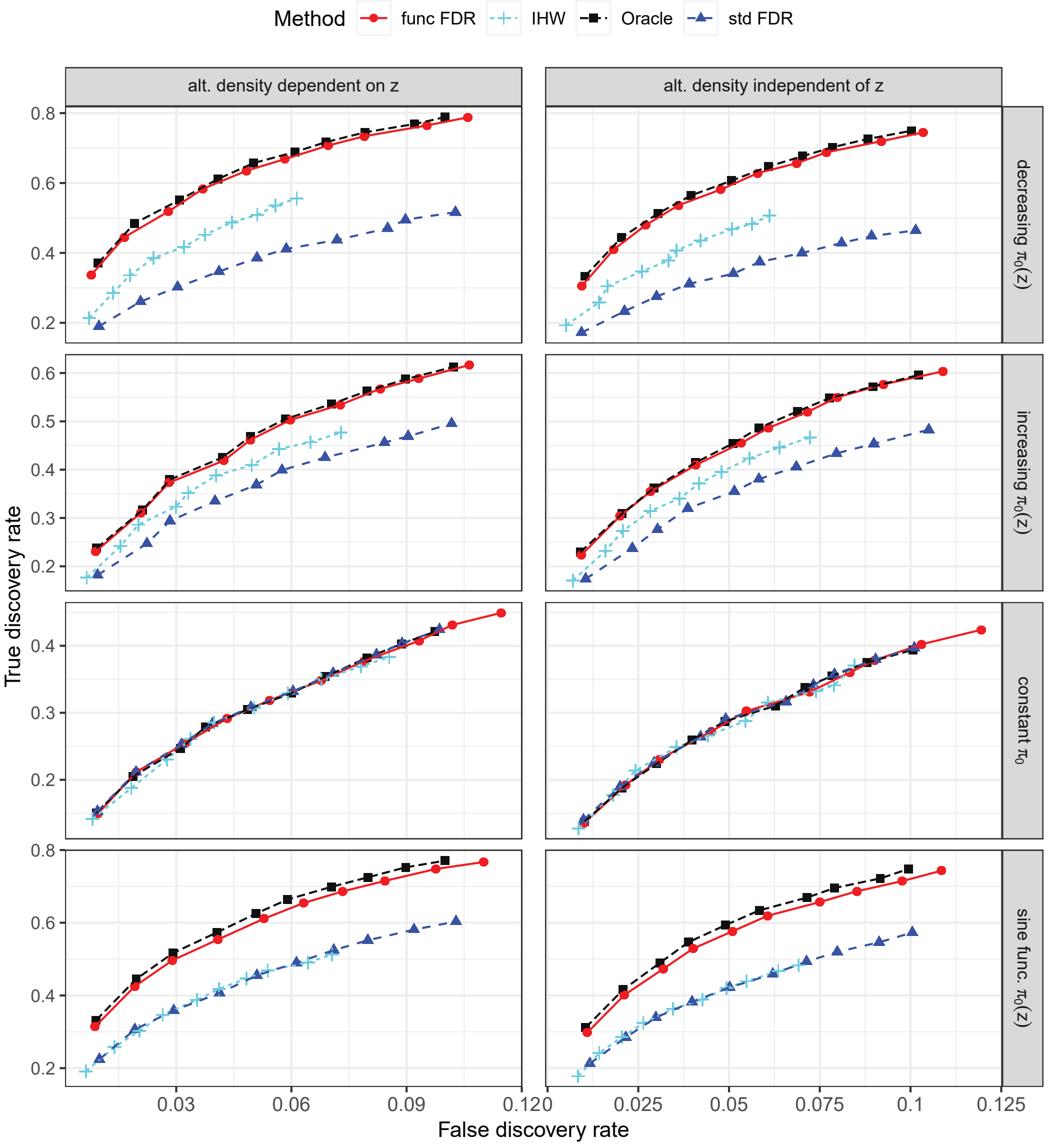

Fig. 1. Performance of the fFDR method (func FDR) against those of the standard FDR method (std FDR) and the "IHW" method, with reference to that of the "Oracle". The points from left to right on each line in each subfigure are successively obtained for q-value cutoffs $\alpha = 0.01, 0.02, \ldots, 0.1$. The column and row plot titles denote the type of $\pi_0(z)$ and alternative density $f_1(p|z)$ utilized. It can be seen that the power of the fFDR method is very close to that of the Oracle and that it is greater than those of the standard FDR method and the IHW method when $\pi_0(z)$ is not a constant.

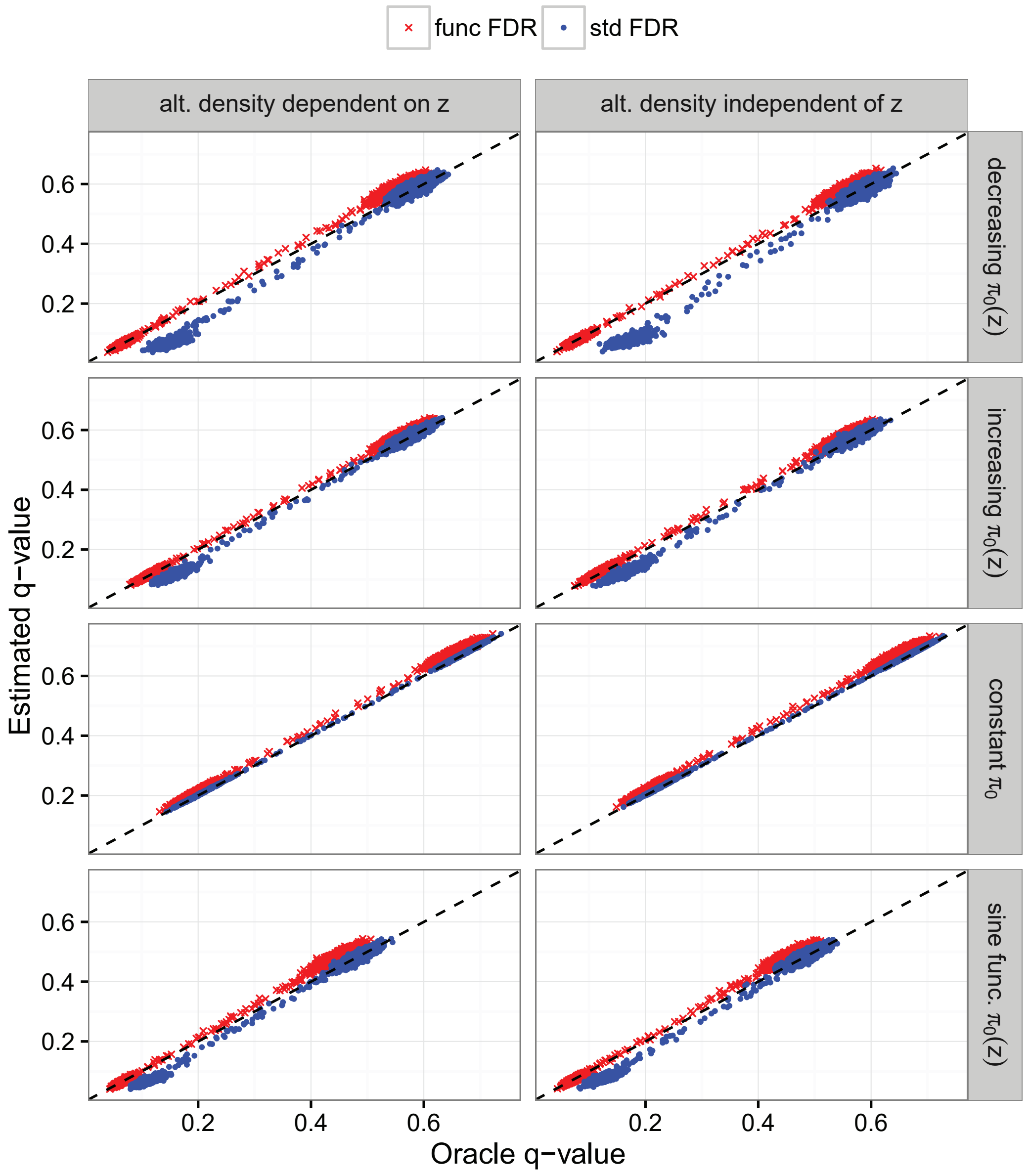

Fig. 2. Estimated q-values versus oracle q-values, where the oracle q-values are computed based on the true underlying model. The dashed line in each plot indicates perfect estimates of the oracle q-values. The column and row plot titles denote the type of $\pi_0(z)$ and alternative density $f_1(p|z)$ utilized. It can be seen that the fFDR method (func FDR) estimates oracle q-values accurately and does so more accurately than the standard FDR method (std FDR).

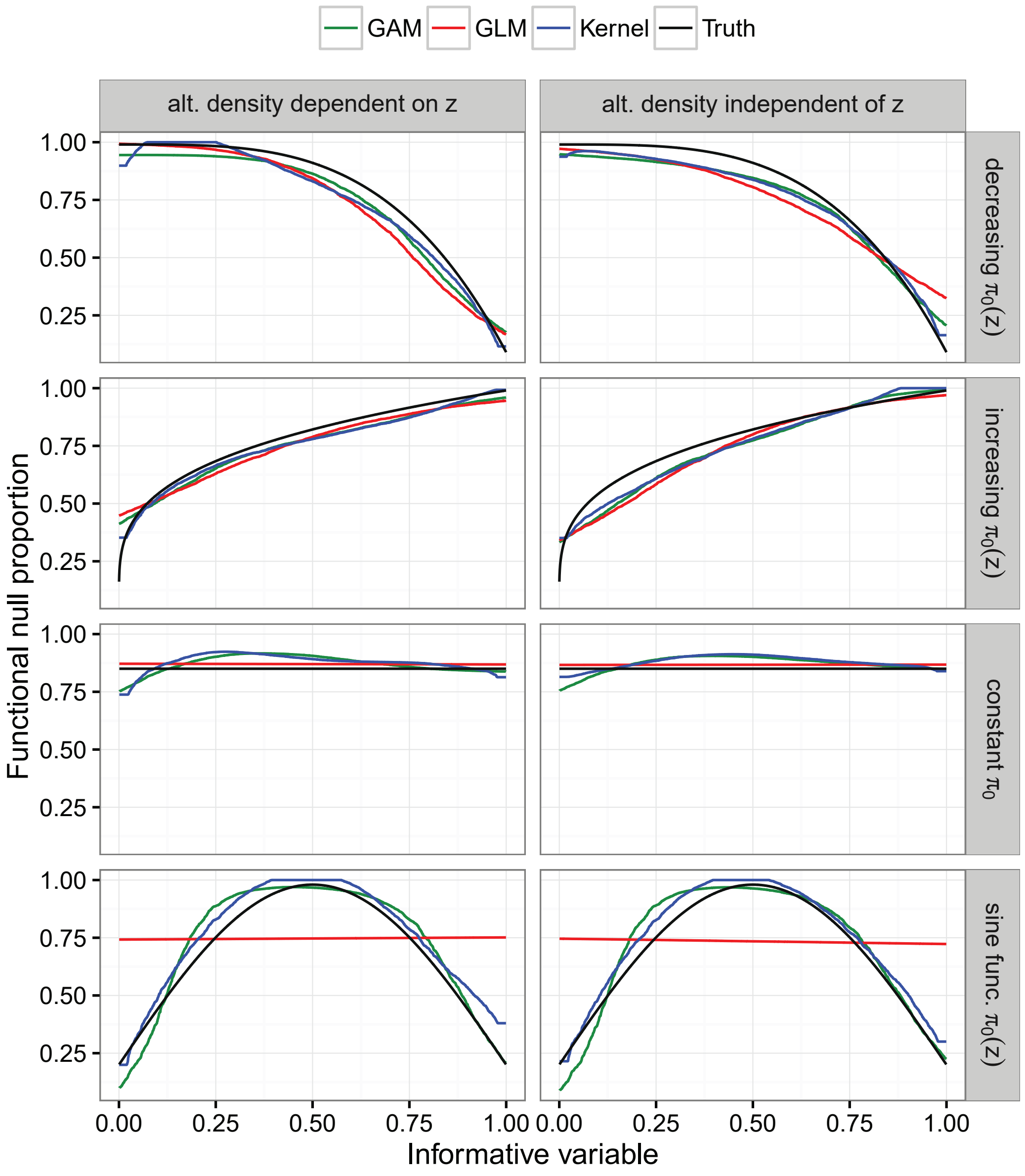

Fig. 3. Performance of the estimator $\hat{\pi}_0(z;\lambda)$ of the functional null proportion $\pi_0(z)$. The column and row plot titles denote the type of $\pi_0(z)$ and alternative density $f_1(p|z)$ utilized.

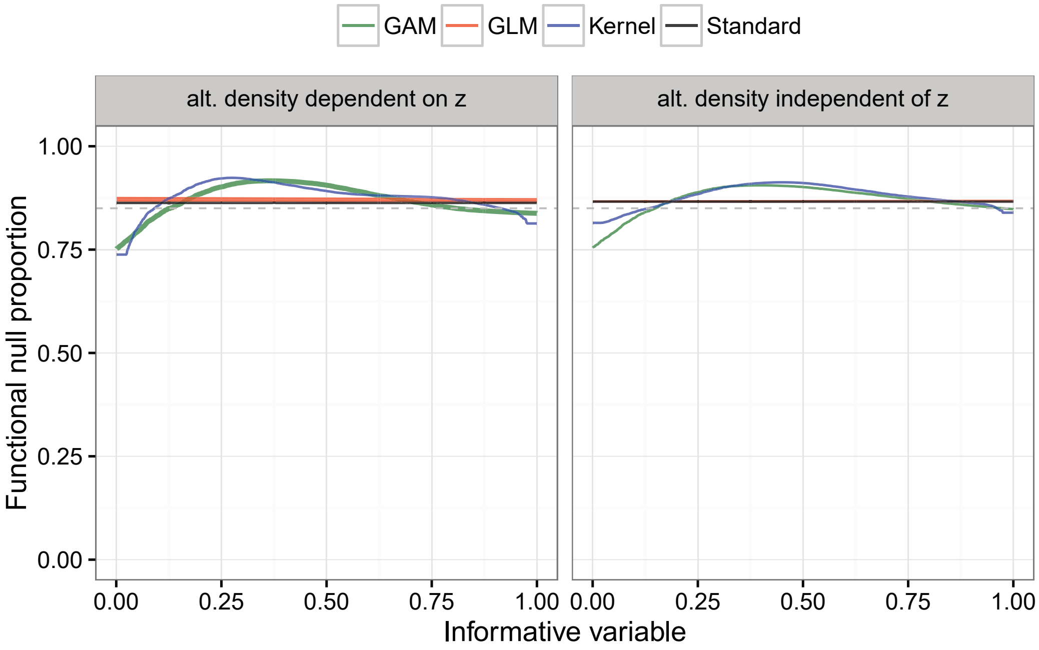

Fig. 4. The estimator $\hat{\pi}_0(z;\lambda)$ when π_0 is constant. The plot titles indicate whether the alternative density $f_1(p|z)$ depends on z or not. In the legend "Standard" refers to the estimate of π_0 provided by the `qvalue` package (Storey et al., 2015), and the dashed line indicates the true value $\pi_0 = 0.85$.

[*Received August 1, 2010; revised October 1, 2010; accepted for publication November 1, 2010*]

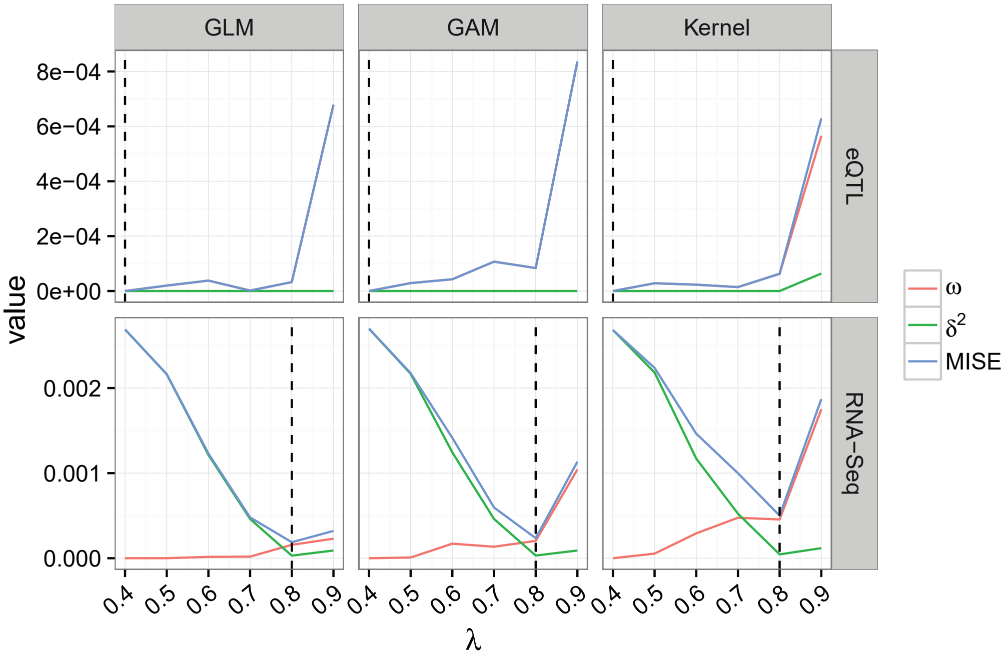

Fig. 5. Determining the tuning parameter λ in the estimator $\hat{\pi}_0(z;\lambda)$ shown in Figure 2 in the main text for the eQTL and RNA-seq analyses. In the legend, ω is the estimated integrated variance defined by (2.5), δ^2 the square of the estimated integrated bias defined by (2.6), and MISE the estimated "mean integrated squared error" defined by (2.7). The chosen $\hat{\lambda}$ minimizes the MISE and is indicated by the vertical dashed line.

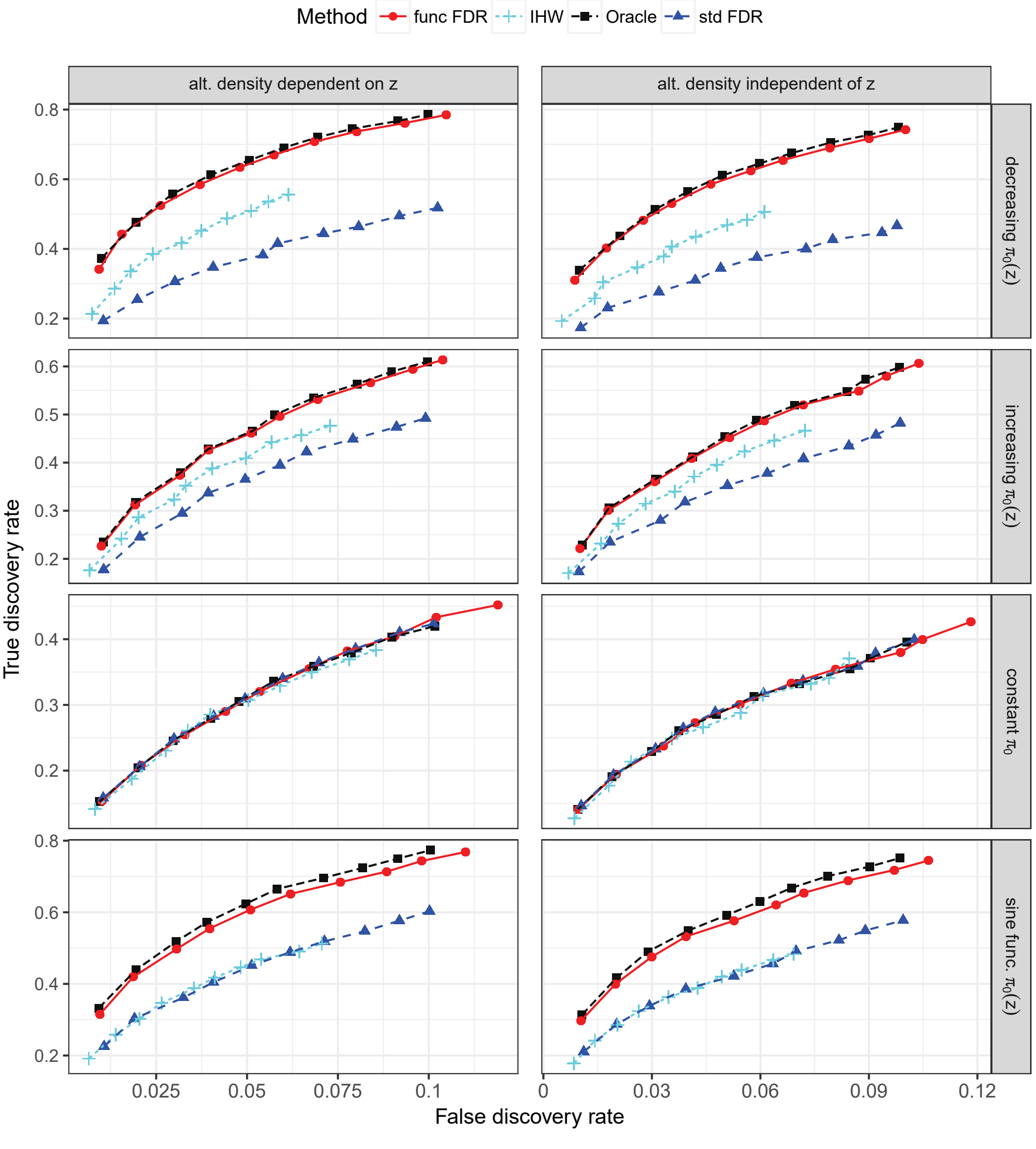

Fig. 6. Performance of the fFDR method (func FDR) against those of the standard FDR method (std FDR) and the "IHW" method, with reference to that of the "Oracle". The points from left to right on each line in each subfigure are successively obtained for q-value cutoffs $\alpha = 0.01, 0.02, \ldots, 0.1$. The column and row plot titles denote the type of $\pi_0(z)$ and alternative density $f_1(p|z)$ utilized.

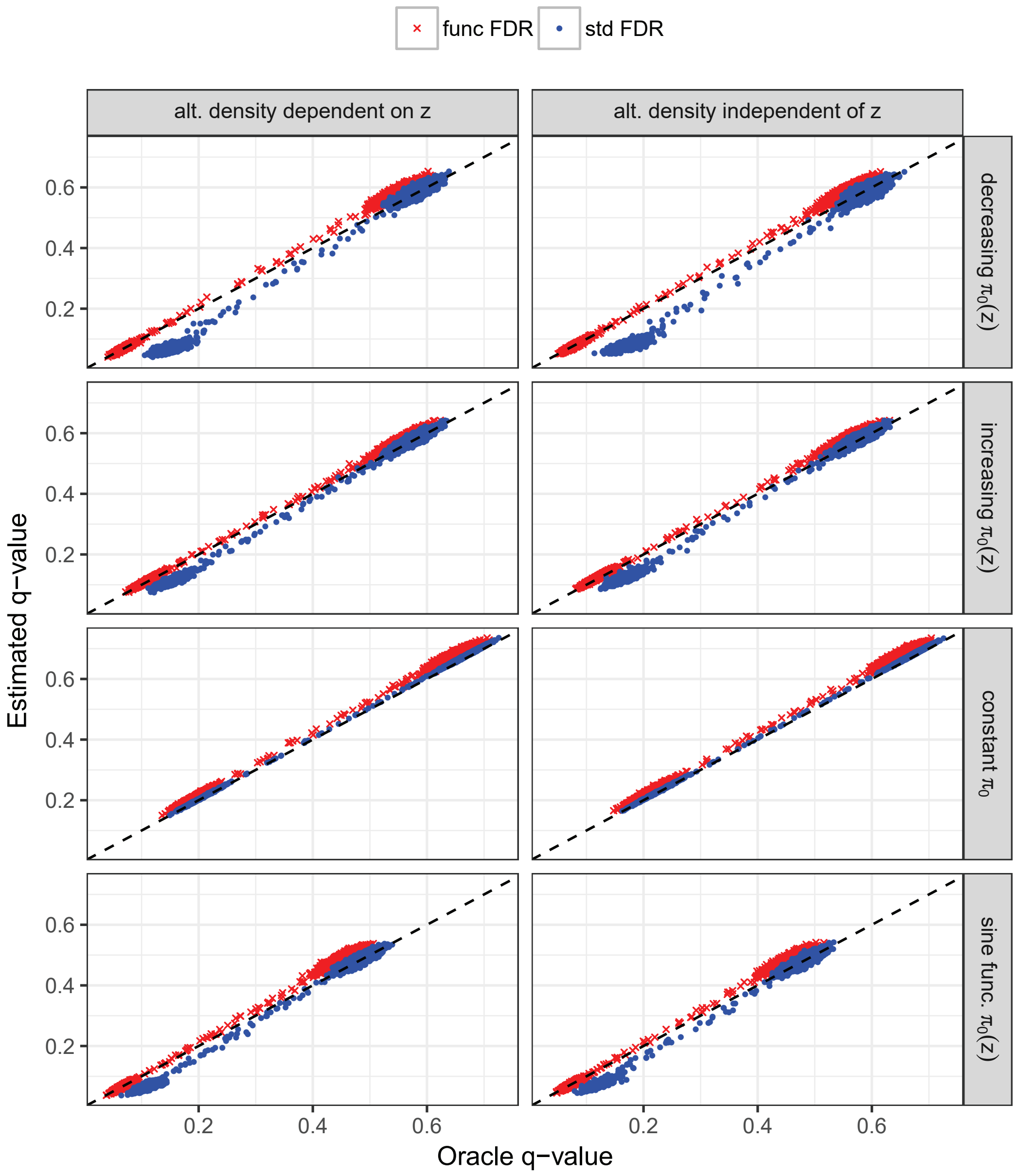

Fig. 7. Estimated q-values versus oracle q-values, where the oracle q-values are computed based on the true underlying model. The dashed line in each plot indicates perfect estimates of the oracle q-values. The column and row plot titles denote the type of $\pi_0(z)$ and alternative density $f_1(p|z)$ utilized.

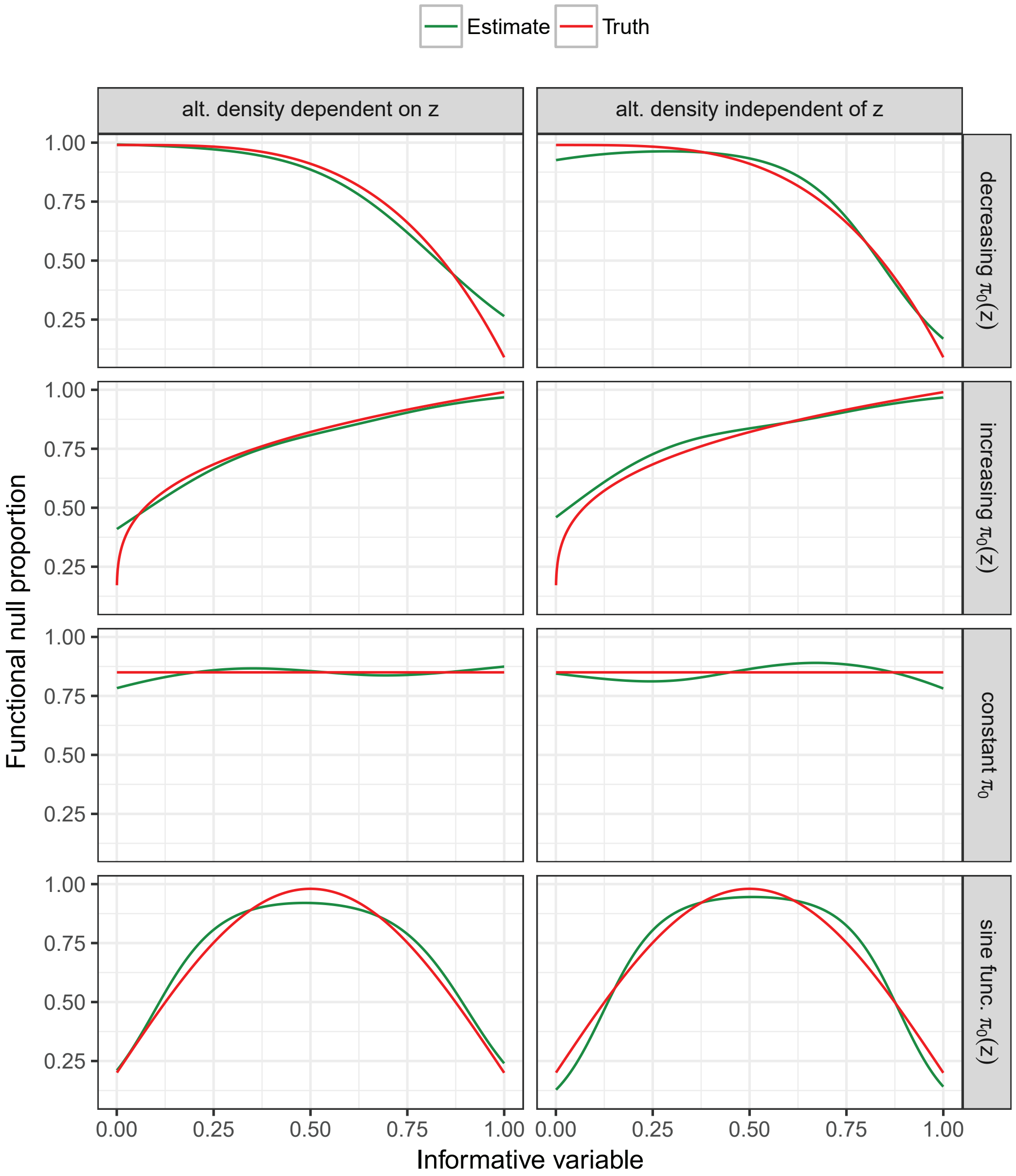

Fig. 8. Performance of the estimator $\hat{\pi}_0(z;\lambda)$ of the functional null proportion $\pi_0(z)$. The column and row plot titles denote the type of $\pi_0(z)$ and alternative density $f_1(p|z)$ utilized. $\hat{\pi}_0(z;\lambda)$ is obtained using the GAM method.

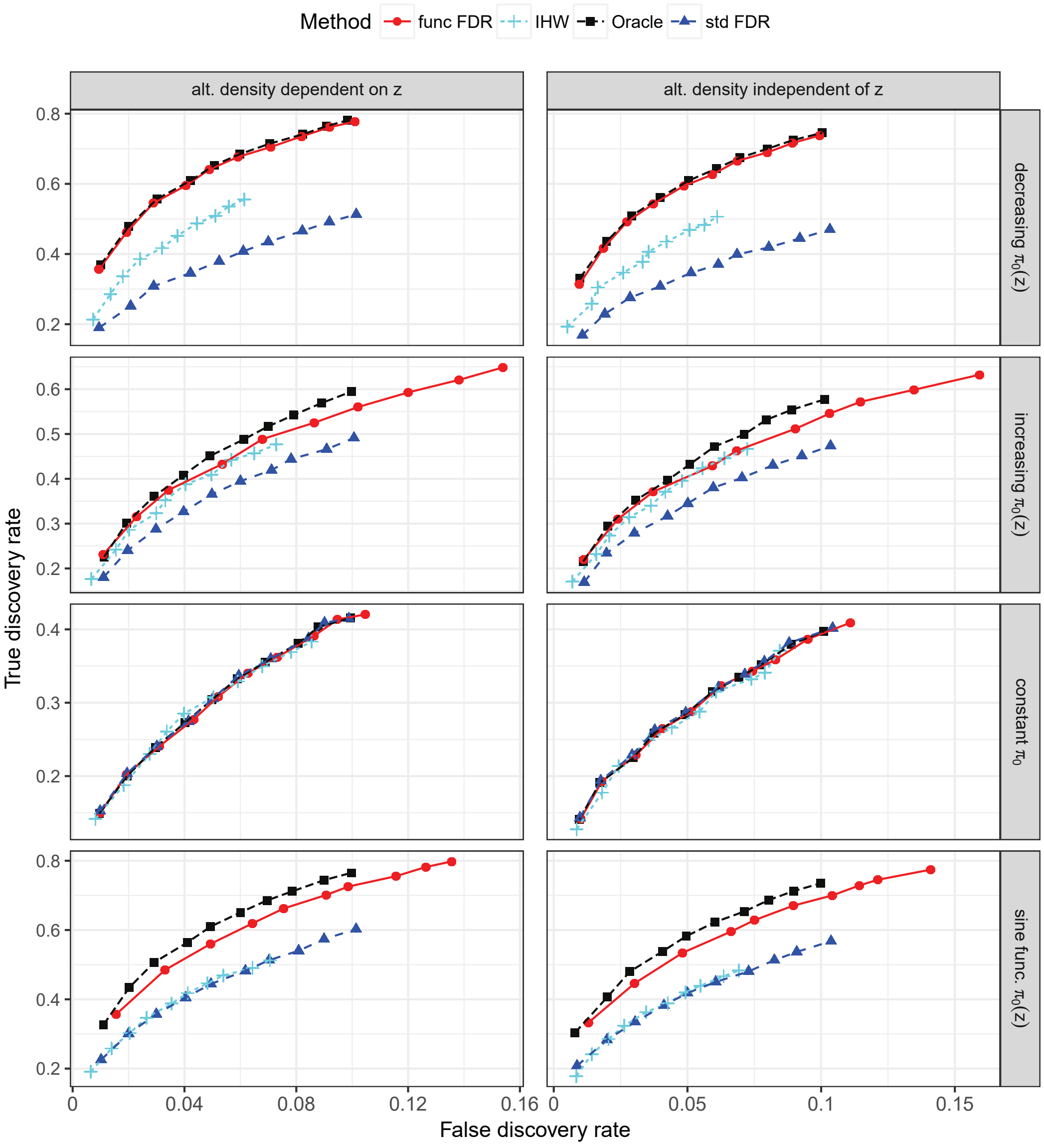

Fig. 9. Performance of the fFDR method (func FDR) against those of the standard FDR method (std FDR) and the "IHW" method, with reference to that of the "Oracle". The points from left to right on each line in each subfigure are successively obtained for q-value cutoffs $\alpha = 0.01, 0.02, \ldots, 0.1$. The column and row plot titles denote the type of $\pi_0(z)$ and alternative density $f_1(p|z)$ utilized.

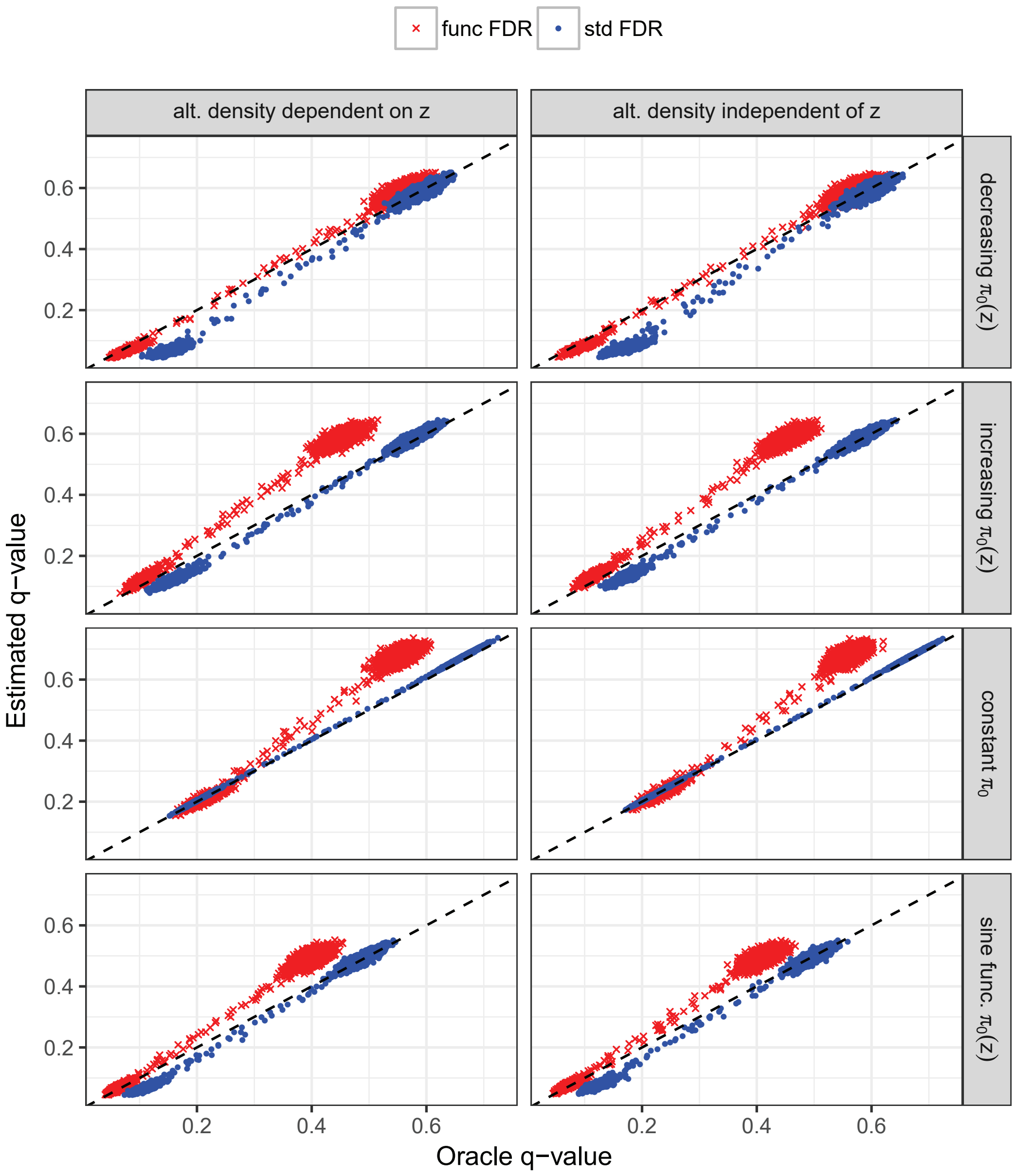

Fig. 10. Estimated q-values versus oracle q-values, where the oracle q-values are computed based on the true underlying model. The dashed line in each plot indicates perfect estimates of the oracle q-values. The column and row plot titles denote the type of $\pi_0(z)$ and alternative density $f_1(p|z)$ utilized.

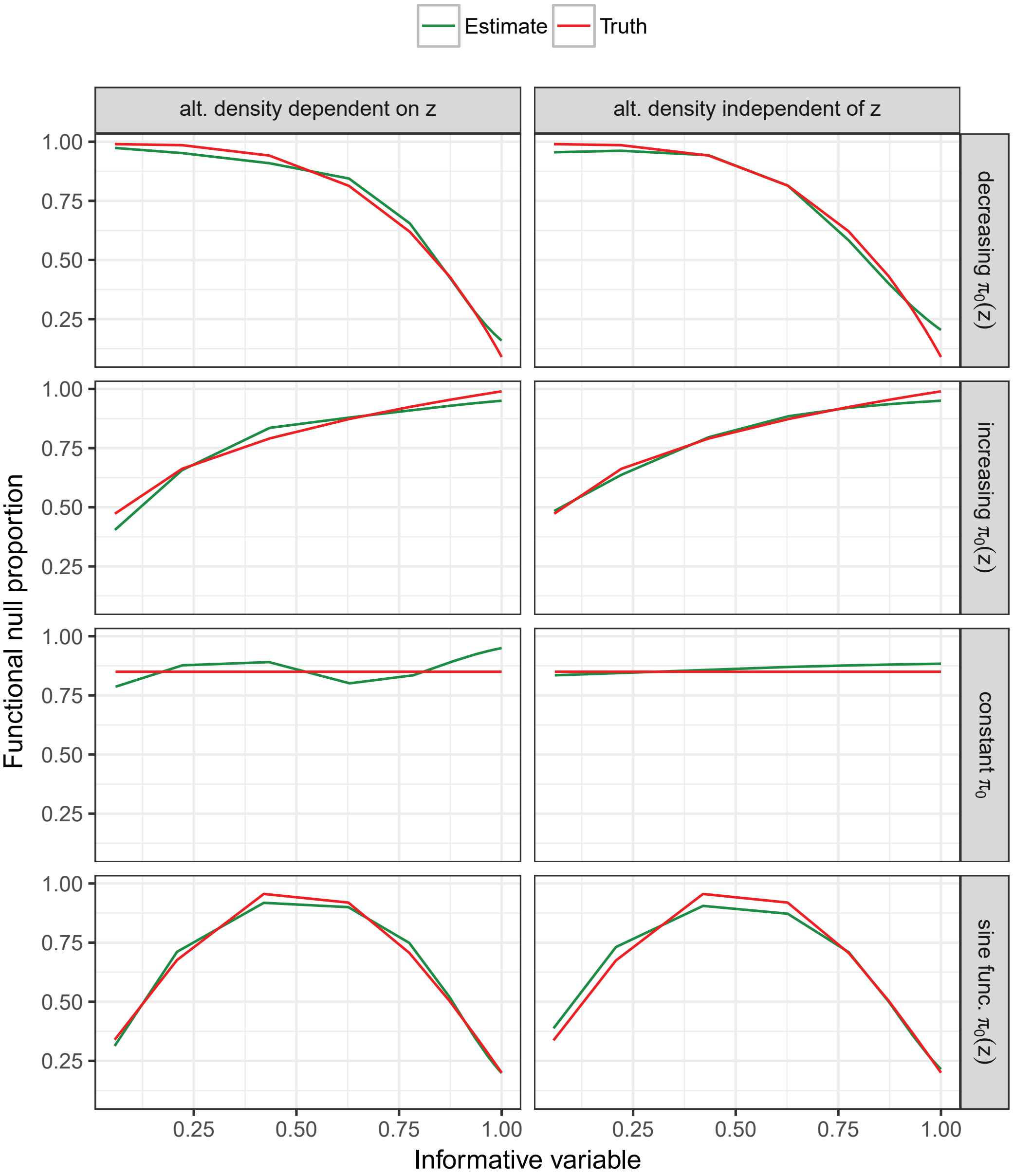

Fig. 11. Performance of the estimator $\hat{\pi}_0(z;\lambda)$ of the functional null proportion $\pi_0(z)$. The column and row plot titles denote the type of $\pi_0(z)$ and alternative density $f_1(p|z)$ utilized. $\hat{\pi}_0(z;\lambda)$ is obtained using the GAM method.
